# Layers to leaves: A suite of modular 3D printed hydroponics components for research and education

**DOI:** 10.1371/journal.pone.0346497

**Published:** 2026-04-29

**Authors:** Ethan A. Shaw, Suraj K. Chandramouli, Michael P. Dzakovich

**Affiliations:** 1 Department of Pediatrics, USDA-ARS Children’s Nutrition Research Center, Baylor College of Medicine, Houston, Texas, United States of America; 2 Department of Biomedical Engineering, University of Houston, Houston, Texas, United States of America; 3 Department of Biosciences, Rice University, Houston, Texas, United States of America; King George's Medical University, INDIA

## Abstract

Hydroponics is a widely utilized technique to precisely control the plant growing environment and maximize productivity. In some cases, hydroponics systems can be expensive and require specialized expertise to build and maintain. Here, we present a suite of 3D-printed devices that can be constructed into a single or double tower hydroponics system of variable height and composition. This system is easily scalable to fit the needs of a user and can be implemented in a variety of research and educational contexts. Components in this suite can be made from inexpensive plastic filament using household-grade 3D printers. The vertical design of these systems allows for users to maximize space-use-efficiency in growth chambers, greenhouses, or even classrooms. We describe the construction of our system and provide example data from a validation study growing spinach (*Spinacia oleracea* L*.*) under different salinity conditions. Data generated by our 3D printed system were comparable to those generated using traditional deep water culture. This suite of 3D printed components can be utilized by researchers and educators alike to capitalize on the benefits of hydroponics in a flexible, budget-friendly way.

## Introduction

3D printing is an emerging form of additive manufacturing that allows for the creation of custom parts tailored to meet the needs of an end user. Although metal-based 3D printers are utilized in high-end industrial manufacturing, most 3D printers available to consumers use inexpensive plastic filament (e.g., polylactic acid; PLA) that is widely available online or in retail stores [1]. Manufacturing advances have allowed the 3D printer industry to generate a range of product lines available at a low cost to consumers; many entry level devices cost around $300. As such, this technology is quickly being adopted in research settings [1–4] as well as incorporated into classroom curriculum to promote STEM education [5–8]. One application of 3D printing is manufacturing modular hydroponic systems that can be adjusted to the needs of the end user without the need for complete refabrication.

Hydroponics is a widely used approach to maximize plant growth, development, and uniformity. Plants grown hydroponically benefit from optimized mineral nutrition, absence of water stress common in field-grown crops, and are often grown in systems that generally control other environmental factors (e.g., vertical farms) [9,10]. While hydroponics has long been adopted by industry to maximize plant yield, it continues to have innumerable applications for research. Mineral nutrition studies can be easily conducted using hydroponics due the customizable nature of nutrient solutions and minimized nutrient adsorption/desorption that commonly occurs with solid substrates and soil [11]. By making mineral nutrition consistent among plants, the effects of plant genotype as well as environmental variables can be studied in relative isolation compared to soil or field-grown plants [12–14]. Hydroponics also accommodates intrinsic labeling using precious radiolabeled or nonradioactive isotopes that would be lost to a solid substrate [11]. These experiments provide deeper insights into nutrient turnover and partitioning as well as their bioavailability in nutritional studies. While there are countless uses for hydroponics in industry and research, this technique is increasingly being utilized as a means of STEM education in school curriculum.

Hydroponics can be utilized in educational settings for a wide range of student ages to provide hands-on learning experiences in plant biology [15–18]. Limited studies indicate that gardening and hydroponics can improve children’s attitudes towards fruit and vegetable consumption [19–22] as well as their perceptions of science, technology, engineering, and math (STEM) disciplines [16,17,23,24]. Similarly, 3D printing is also quickly becoming integrated into school curriculum for elementary school to university aged individuals [5–8]. There is enormous potential to leverage 3D printing to create modular, user-friendly hydroponics systems for both research and educational purposes.

We sought to develop a suite of 3D printed objects that can be assembled into a cost-effective and modular hydroponics system. As the components are freely available, this work represents an effort to democratize hydroponics to both the plant biology community and the public. Components from this suite of models can also be used independently of the systems we designed (e.g., plant cups) for other hydroponics devices or other applications altogether. While the components are meant to work in concert with one another, these objects can serve as scaffolds for in any number of downstream applications that users are free to adapt. Here, we describe the components of the system, its construction, a proof of concept study with spinach (*Spinacia oleracea* L.), and establish that this system performs comparably to traditional deep water culture systems.

## Materials and methods

### System description

The modular hydroponics system consists of three main parts: 1) the nutrient solution bin; 2) one or more 3D printed tower(s); 3) the 3D printed lid. The system is designed to support either a Single or Double Tower configuration; maximizing flexibility for various end users (e.g., researchers, educators) while remaining cost-effective using 3D printing (Tables 1-2). Components are easily interchangeable, allowing users to adapt or integrate parts into other hydroponic setups as needed. Files for 3D components can be found in the NIH3D database for the Single (https://3d.nih.gov/entries/3DPX-021941) and Double (https://3d.nih.gov/entries/3DPX-021942) Tower systems [25,26].

**Table 1:** Summary of major hobbyist 3D printers. Standardized build volumes and whether each model can accommodate the 3D printed hydroponics system’s largest required print profile (177.29 x 177.29 x 144.28 mm) object.

Printer Name	Bed Size (mm)	Compatible with 3D Printed Hydroponics Suite?
Creality Ender-3 Series	220 x 220 x 250	✓
Anycubic Kobra Series	250 x 250 x 250	✓
Flashforge Adventurer Series	220 x 220 x 250	✓
Prusa MK3/MK4 Series	250 x 210 x 210	✓
Bambu Lab A1	256 x 256 x 256	✓
Bambu Lab A1 Mini	180 x 180 x 180	✓
Bambu Lab P1 Series	256 x 256 x 256	✓
Creality K1/ K1C	220 x 220 x 250	✓
QIDI X-Plus/ Plus4	225 x 225 x 230	✓
Artillery Sidewinder X-Series	300 x 300 x 400	✓

**Table 2:** Tower modules for a standard single tower system.

Tower Modules – Single Tower System (3x1)						
Part #	File Name	Part Name	Quantity per System^a^	Mass per Part (g)	Mass per System (g)	Cost per System ($USD)
1	4-Cup_Planting_Module.stl	4-cup ST Planting Module	3	216.02	648.06	$12.31
2	Tower_Lid.stl	Tower Lid	1	39.25	39.25	$0.75
3	Tower_Distributor.stl	Tower Distributor	1	82.91	82.91	$1.58
4	Stream_Breaker.stl	Stream Breaker	1	7.75	7.75	$0.15
5	Tower_Short_Spacer.stl	Tower Short Spacer	2	55.23	110.46	$2.10
6	Standard_Pot.stl	Standard Pot	12	25.2	302.4	$5.75
7	Reservoir_Tower_Adaptor.stl	Tower Adaptor	1	56.31	56.31	$1.07

^a^Assuming a system composition of 3 modules. Users may alter the number of modules/height of their system according to their own needs.

### System methodology: Cost framework, print compatibility, and material summary

This is a budget-conscious, modular parts ecosystem, where overall cost-effectiveness has been factored, and the profiles of each piece have been tailored to be compatible within the bed-sizes of most widely available “hobbyist” 3D printers (Table 1). Baseline pricing assumes ~20% infill, 0.2 mm layer height, grid infill, and ~$19 per 1 kg filament spool (Tables 2–6). We note that cost estimates are based on prices found through various online vendors on December of 2025 and estimated system parts for both the Single and Double Towers systems are assuming the user wants a 3-module tall system. Users are free to add or subtract modules as needed. The parts sheet (Table 7) and total external materials list (Table 8) provide transparent, approximate line-item costs for construction of the default Single Tower system. In practice, totals may vary with infill density, filament brand, and tool availability. However, the cumulative expenditure remains lower than comparable prefabricated hydroponic units while preserving flexibility for user-specific modifications.

**Table 3:** Lid modules for the single tower system.

Lid Modules – Single Tower System (3x1)						
Part #	File Name	Part Name	Quantity per System	Mass per Part (g)	Mass per System (g)	Cost per System ($USD)
8	Reservoir_Spacer_Long_Edge.stl	Reservoir Spacer Long Edge	2	17.17	34.34	$0.65
9	Reservoir_Lid_Left.stl	Reservoir Lid Left	2	163.91	327.82	$6.23
10	Reservoir_Lid_Right_Access_Hole.stl	Reservoir Lid Right Access Hole	1	150.81	150.81	$2.87
11	Reservoir_Lid_Right.stl	Reservoir Lid Right	1	164.28	164.28	$3.12
12	Reservoir_Spacer_Short_Edge.stl	Reservoir Spacer Short Edge	2	9.18	18.36	$0.35
13	Access_Hole_Cap	Access Hole Cap	1	25.00	25.00	$0.48
14	NA	M5 Screw and nut – 25 mm	6	NA	NA	NA

**Table 4:** Tower modules for the double tower system.

Tower Modules – Double Tower System (3x2)						
Part #	File Name	Part Name	Quantity per System^a^	Mass per Part (g)	Mass per System (g)	Cost per System ($USD)
24	DT_4-Cup_Planting_Module.stl	4-cup DT Planting Module	12	213.1	2557.20	$48.59
2	Tower_Lid.stl	Tower Lid	2	39.25	2557.20	$48.59
3	Tower_Distributor.stl	Tower Distributor	2	82.91	78.50	$1.49
4	Stream_Breaker.stl	Stream Breaker	2	7.75	165.82	$3.15
5	Tower_Short_Spacer.stl	Tower Short Spacer	4	55.23	15.50	$0.29
6	Standard_Pot.stl	Standard Pot	12	25.2	220.92	$4.20
7	Reservoir_Tower_Adaptor.stl	Tower Adaptor	2	56.31	302.40	$5.75

^a^Assuming a system composition of 3 modules. Users may alter the number of modules/height of their system according to their own needs.

**Table 5:** Lid modules for the double tower system.

Lid Modules – Double Tower System (3x2)						
Part #	File Name	Part Name	Quantity per System	Mass per Part (g)	Mass per System (g)	Cost per System ($USD)
13	Access_Hole_Cap	Access Hole Cap	1	25.00	25.00	$0.48
18	Reservoir_Lid_DT_Right.stl	Reservoir Lid DT Right	1	110.42	110.42	$2.10
19	Reservoir_Lid_DT_Left.stl	Reservoir Lid DT Left	2	110.15	220.30	$4.19
20	DT_Spacer_Long_Edge.stl	DT Spacer Long Edge	4	9.83	39.32	$0.75
21	DT_Spacer_Short_Edge.stl	DT Spacer Short Edge	2	9.39	18.78	$0.36
22	Reservoir_Lid_DT_Middle.stl	Reservoir Lid DT Middle	2	97.18	194.36	$3.69
23	DT_Spacer_Middle_Edge.stl	DT Spacer Middle	1	3.26	3.26	$0.06
25	Reservoir_Lid_DT_Right_Access_Hol.stl	Reservoir Lid DT Right Access Hole	1	97.10	97.10	$1.84
14	NA	M5 Screw and nut – 25 mm	7	NA	NA	NA

**Table 6:** Part variations for all tower systems.

Part Variations – Tower Modules						
Part #	File Name	Part Name	Quantity per System	Mass per Part (g)	Mass per System (g)	Cost per System ($USD)
15	Short_Pot.stl	Short Pot	NA	14.09	NA	$0.27
16	ST_3-Cup_Planting_Module.stl	3-cup ST Planting Module	NA	196.59	NA	$3.74
17	ST_5-Cup_Planting_Module.stl	5-cup ST Planting Module	NA	231.49	NA	$4.40
26	True_Standard_Pot.stl	True Standard Pot	NA	26.7	NA	$0.51
27	DT_3-Cup_Planting_Module.stl	3-cup DT Planting Module	NA	194.53	NA	$3.70
28	Tower_Tall_Spacer.stl	Tower Tall Spacer	NA	126.25	NA	$2.40

**Table 7:** External components and materials for practicum 3.

Material Name	Item Quantity per System	Typical Quantity Sold	Cost per Purchase ($USD)	Effective Cost per System ($USD)
3D Printer (PLA-compatible)	1	1 unit	$172.00	$172.00
M5 Screws (25 mm length)	8	100-pack	$12.49	$1.00
M5 Nuts	8	100-pack	$6.49	$0.52
M5 Allen Wrench	1	1 set (metric 2–10 mm)	$24.99	$24.99
Adjustable Wrench	1	1 tool	$13.98	$13.98
Silicon Sealant	~2 oz	1 tube (10 oz)	$6.78	$1.36
Silicon Sealant Extruder/Tube Gun	1	1 tool	$19.95	$19.95
Aluminum Foil	~5 ft	1 roll (75 ft)	$12.97	$0.86
Box Cutter/ Similar Cutting Tool	1	1 tool	$10.99	$10.99
Flexible Vinyl Tubing (½″ ID)	~5 ft	10 ft roll	$8.99	$4.50
Hydroponic Pump	1	1 unit	^**a**^$25.99	$25.99
500-gram Weight	1	1 pc	^**a**^$11.99	$11.99
Storage Bin (ULINE S-20588GR or similar)	1	1 bin (~20 L)	^**a**^$19.00	$19.00
Rotary Tool with Cutoff Disc	1	1 set	$50.00	$50.00
Foam Weather Stripping (¾″ × 7⁄16″ thick)	~2.5 ft	1 roll (10 ft)	$8.59	$21.48
Soft Cloth or Sponge	1	3-pack	$3.49	$1.16
Dark Opaque Paint	1	1 can (12 oz)	$6.97	$6.97
PVC T-Joint (½″ ID)	1	5-pack	$5.99	$1.20

^a^Indicates per-system costs incurred for each additional system built.

**Table 8:** Part totals required for initial and subsequent assemblies.

Total Price for the Assembly of Single Tower (ST) System	
Material Category	Total ($USD)
Tower Modules – Single Tower System (3x1) [Table 2]	$23.70
Lid Modules – Single Tower System (3x1) [Table 3]	$13.69
Total Material Cost ($) [Table 2 + 3]	^**a**^$37.39
Purchased Components and Materials [Table 6]	$408.69
TOTAL COST ($) [INITIAL]	
$446.08	
**TOTAL COST ($) [PER. ADDITIONAL SYSTEM]**	
**•** $94.37	

^a^Single tower system totals derived from Tables 1, 2, and 6 assuming a three-tier tower. Costs will vary if a user wants the tower to be taller or shorter.

### Nutrient solution bin and pump

The hydroponics system was designed to work with ULINE S-20588GR 20L Round Trip Totes (ULINE; Pleasant Prairie, Wisconsin, United States). These bins, measuring 21.8” × 15.2” × 9.3,” are made of high-density polyethylene, a durable, chemically inert material, and are rated to support up to 70 lbs. To inhibit algal growth, a dark-colored bin (e.g., gray) was used to minimize light penetration into the nutrient solution.

Each bin also houses an ActiveAqua Submersible Pump 250 (Hydrofarm; Petaluma, California, USA) located in the center, which circulates nutrient solution from the reservoir to the top of the planting towers. Similar pumps, or even external pumps, may be utilized by the user at their discretion. Structural modifications to the system would need to be made to accommodate an external pump.

### 3D printed towers

**Fig 1:**
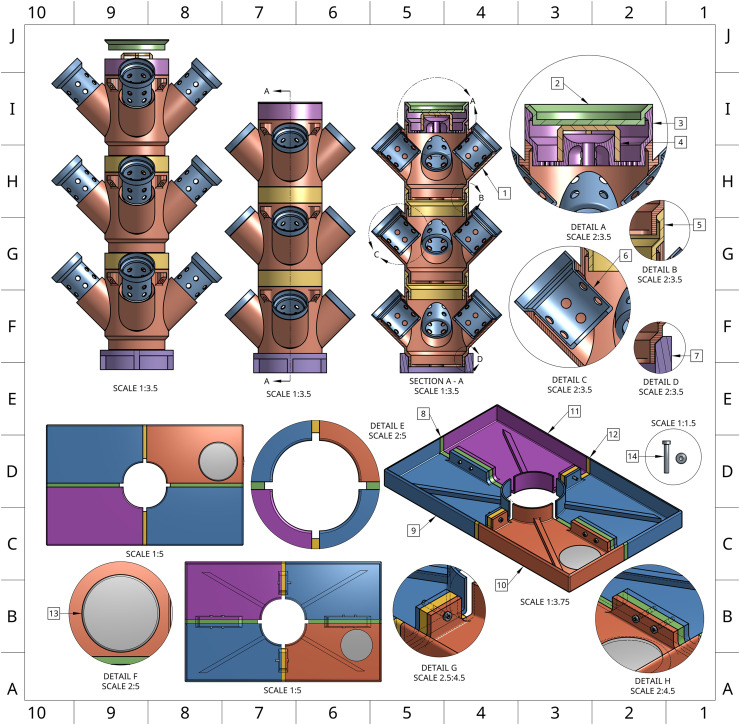
Multi-perspective rendering of the Single Tower system highlighting lid and tower components. An interactive 3D rendering of the Single Tower system (https://sketchfab.com/3d-models/one-tower-enviroment-696d529bdf3249bb8fe916f2aff49538) is available for close examination of parts in the context of a completed system.

**Fig 2:**
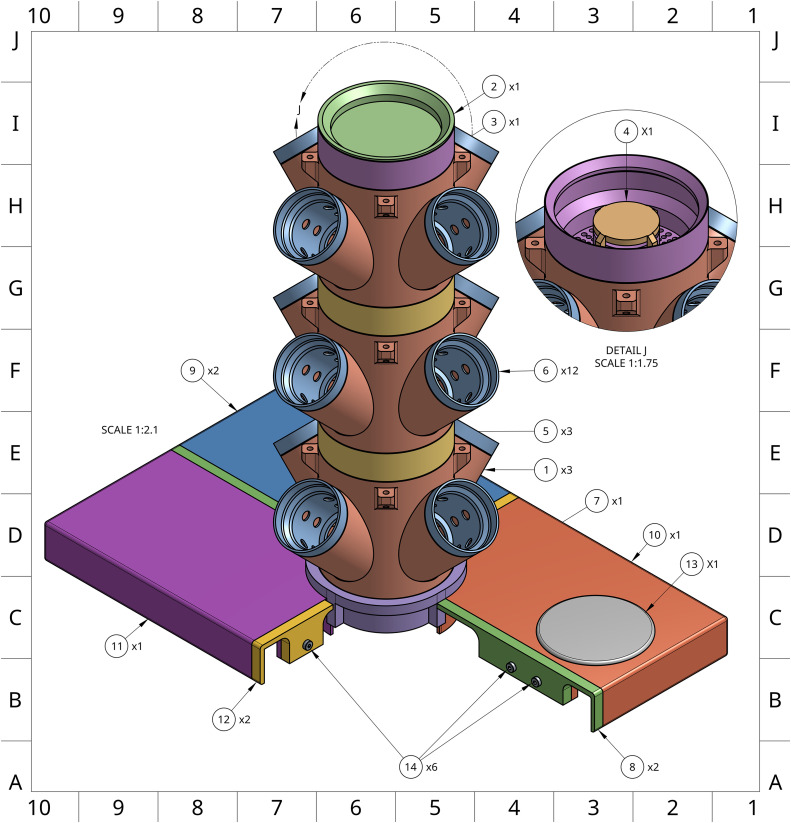
Detailed rendering of the Single Tower system showing required components necessary for assembly except for one lid piece for a clearer view of the tower-lid interface. An interactive 3D rendering of the Single Tower system (https://sketchfab.com/3d-models/one-tower-enviroment-696d529bdf3249bb8fe916f2aff49538) is available for close examination of parts in the context of a completed system.

**Fig 3:**
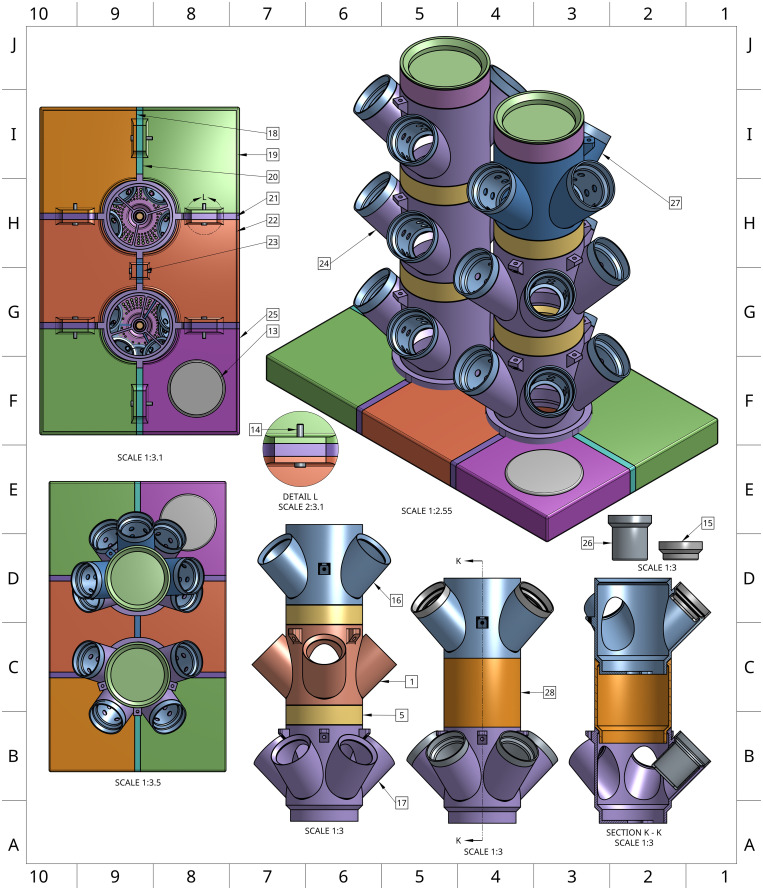
Multi-perspective rendering of the assembled Double Tower system and unique Double Tower components. An interactive 3D rendering of the Double Tower system (https://sketchfab.com/3d-models/two-tower-hydroponics-assembly-variant-models-d80f772011a44d1e928962e8bdacf65a) is available for close examination of parts in the context of a completed system.

To improve growing capacity beyond what a horizontal growth-system allows, our system capitalizes on vertically stacked planting towers (Figs 1 and 2). Each tower is built using modular 4-cup units, available in 3- and 5-cup variants, which are stacked with spacer modules in either standard or extended lengths (Fig 3 parts 5 and 28). These variable planting modules have a finite number of spaces for plant cups that allow for 2” neoprene cloning collars to support plants throughout their lifecycle. Single-tower plant modules, cups, spacers, and stream breaker, are heavily modified from Thingiverse user ‘boundarycondition’ [27].

All modules are designed with a central channel (Fig 1) for routing ½” internal-diameter flexible vinyl tubing, which runs up to a final distributor at the top of the tower. This tubing is secured by the distributor’s barbed connector, and a stream breaker is installed over the distributor opening to diffuse flow (Fig 2). The distributor is then capped and recommended to be held in place with a small (~500 g) weight on the lid or sealed with silicone. Once transported to the top of the tower, the nutrient solution exits through a radial array of drainage holes, evenly cascading down through the tower, irrigating the roots, and returning to the reservoir. This circulation also promotes oxygenation of the rhizosphere to encourage healthy plant growth.

### 3D printed lid

To interface the towers with the ULINE totes, a custom multi-part lid was designed, consisting of modular lid panels, spacer components, and an access cap (Fig 2). These parts were designed as singular components to allow for sizing compatibility with virtually any 3D printer. These parts are secured using M5 screws and nuts (Fig 1, Detail G,H). This bolting configuration, in conjunction with the radially symmetrical tower adaptor (Fig 1, Section A-A), ensures mechanical stability under the compressive force of a loaded tower. To minimize potential leaks and reduce the risk of microbial contamination, all seams are sealed with silicone and optionally wrapped in foil to block passive light transmission.

### Double tower design

To accommodate environments with limited vertical clearance, a Double Tower version of the system was developed (Fig 3; Tables 4–5). This design allows two towers to be used within the same footprint as a single tower, maximizing use of both horizontal and vertical space. The lid was modified to support two towers, and specialized 3- and 4-cup modules were designed to ensure adequate light exposure for all plants. To maintain equal distribution between towers, the system uses a T-junction fitting at the pump outlet to split flow into two channels. Each vinyl tubing line is extended by an additional ~25 cm to accommodate the separate vertical stacks, allowing a single pump to serve both towers.

### Print specification

When printing parts for assembly, we recommend at least 20% infill, ≤ 0.2 mm layer height, and a “grid” infill-pattern to ensure that the system and its components are structurally sound enough to support the tower modules and plants. Parts were fabricated using a Prusa MK3S + , which features a standard bedspace of 250 × 210 × 210 mm. Using a printer with these dimensions or larger ensures that parts can be printed as single pieces rather than being bifurcated due to size limitations, and the user should also determine the most suitable print orientation to ensure each part fits properly within the available build volume. While our 3D components were printed using PLA, other filament materials such as polyethylene terephthalate glycol (PETG) may provide superior chemical stability and waterproofing for some applications.

### Planting module compatibility

Planting modules are cross-compatible within their intended orientation: Single Tower or Double Tower components can be installed in any order or rotated in any direction. However, while modules designed for the Double Tower are compatible with the Single Tower setup, it is not recommended to use Single Tower modules in the Double Tower configuration. Single tower modules do not allow for sufficient space near each plant when installed in a double tower configuration.

### Basic sizing overview

**Fig 4:**
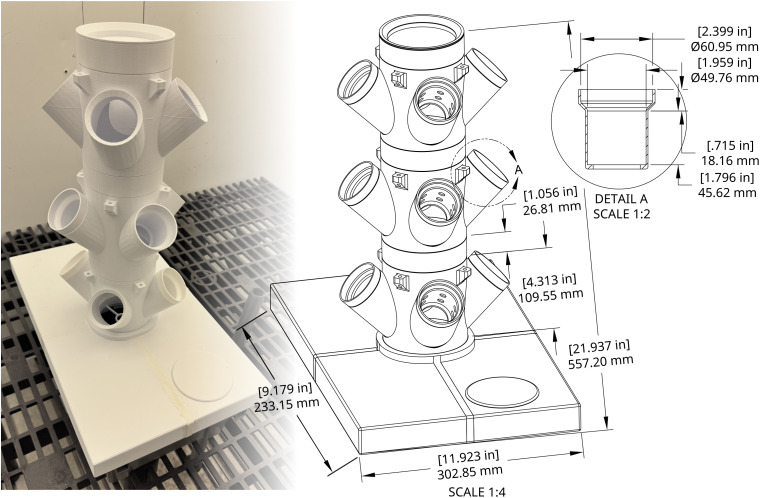
Photograph of an assembled Single Tower system with accompanying rendering showing dimensions of each component.

**Fig 5:**
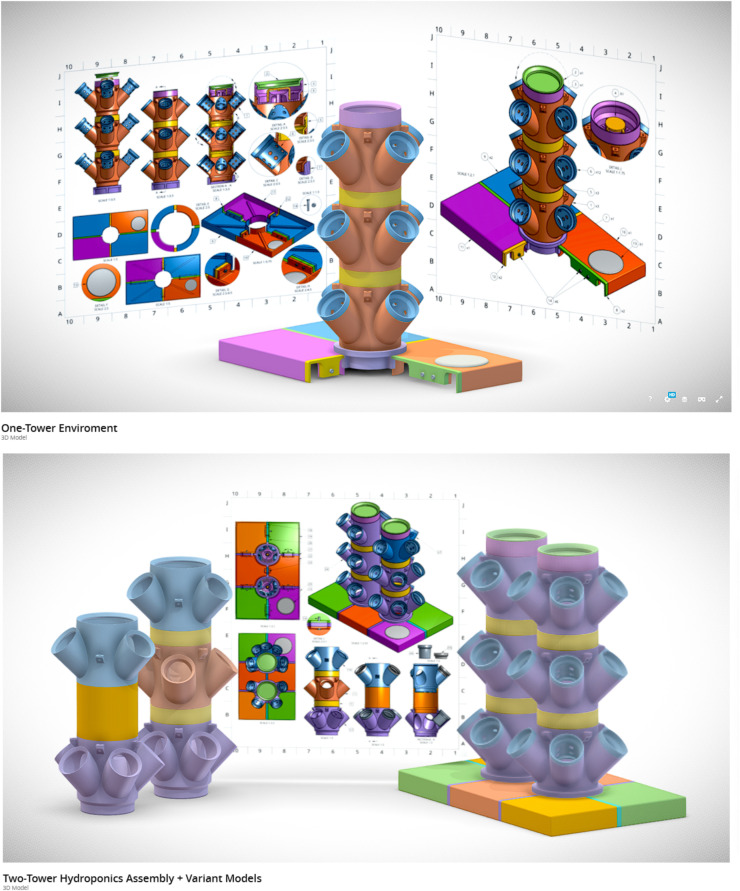
Preview display of the interactive render environments for the Single and Double Tower Assembly variations. An interactive 3D rendering of the Single Tower system (https://sketchfab.com/3d-models/one-tower-enviroment-696d529bdf3249bb8fe916f2aff49538). An interactive 3D rendering of the Double Tower system (https://sketchfab.com/3d-models/two-tower-hydroponics-assembly-variant-models-d80f772011a44d1e928962e8bdacf65a).

When considering location and space limitation for your assembled device, refer to Fig 4, which shows all major dimensions for a standard Single Tower setup (three planting/spacer modules stacked). These sizing dimensions remain the same for the Double Tower variant, as both versions share the same height, width, and length. An interactive 3D rendering of the Single Tower system (Fig 5; https://sketchfab.com/3d-models/one-tower-enviroment-696d529bdf3249bb8fe916f2aff49538) and Double Tower system (Fig 5; https://sketchfab.com/3d-models/two-tower-hydroponics-assembly-variant-models-d80f772011a44d1e928962e8bdacf65a) are available for close examination of parts in the context of a completed system.

### Core device assembly

For details on device assembly please refer to the protocol document on Protocol.IO and included in the Supporting Information (S1 File). Variations for plant cups, planting modules, and spacers that have alternative designs and dimensions are listed in Table 6. The protocol described in this peer-reviewed article is published on protocols.io (dx.doi.org/10.17504/protocols.io.e6nvw4pw9lmk/v2; Supporting Information) and is included for printing purposes as S1 File.

### Nutrient solution considerations

This hydroponics system is designed to function with 15 L of solution in the reservoir. Lower volumes of solution may result in incomplete submersion of the hydroponics pump and would necessitate more frequent changes as plants matured. For our experiment we used Hoagland’s No. 1 nutrient solution [28,29]. However, any hydroponics solution is foreseeably compatible with this system. We recommend conducting preliminary experiments with the crops of interest to determine the optimal frequency by which the nutrient solution is replaced.

### Pump considerations

We used an ActiveAqua Submersible Pump 250 for our experiment. However, any submersible hydroponics pump will work provided it has sufficient power to carry nutrient solution to the top of the tower and can be connected to the ½” inner-diameter tubing. Furthermore, we adjusted the flow rate of the pumps to approximately 200 gallons per hour. This flow rate is likely sufficient for most leafy greens, but may need to be optimized for other crops by the end user. Alternatives such as external pumps or remoting the submersible pump to an additional container using a standpipe may be necessary alternatives for long duration experiments with dense-rooting plant species.

### Planting considerations

We recommend limiting plant selection for this hydroponics system to leafy greens, herbs, and small fruiting crops. One of the limitations of a vertical drip tower design is its relative inability to accommodate plants with substantial taproots (e.g., carrots, turnips, radishes) due to the constraints in plant cup diameter and internal sizing of the tower. Growers should take care to avoid selection of plants with taproots or tuberous roots to ensure they do not encounter challenges with damage to the system and difficult removal of plants after use. Given the modular qualities of our system, users can modify parts as needed to accommodate a wider breadth of plants if desired. The dimensions of our plant cups can be re-scaled for plants with taproots and modified to accommodate solid media, such as expanded clay pellets, that could better support the development of taproots or underground stems in horizontal hydroponics systems. When planting seedlings for use in our vertical tower format, plants should be placed into 2” neoprene collars or a media, such as expanded clay pellets, to ensure they do not shift out of position. Our recommendation is to use a slant board system and transplant seedlings into neoprene collars and install into plant cups after cotyledon emergence as well described by Langenfeld and Bugbee [30,31]. A 3D printed slant board mount can be found here (https://3d.nih.gov/entries/3DPX-021396) [32].We found that direct seeding into neoprene collars without pre-germinating on slant boards resulted in inconsistent emergence and growth.

### Sanitization considerations

During the development of this system, we adopted sanitization procedures before and after each use of the hydroponics system in order to reduce microbial growth in the system. Prior to use, all components of the hydroponics system including pumps and tubing were soaked in 0.05% bleach (sodium hypochlorite) solution for 1 hour, thoroughly rinsed with deionized water afterwards, and left to dry completely. After use, all system components were washed with soap and warm water using a soft sponge and again immersed in 0.05% bleach solution for 1 hour. Components were then rinsed with deionized water before being dried completely prior to storage. We recommend that users adopt some form of sanitization procedure to help minimize the spread of microbial disease during system use. All sanitization protocols should include the thorough mechanical cleaning of parts using soap and water to remove physical debris and microorganism biofilms. Care should be taken to use soft implements to avoid creating scratches in the plastic bin where microorganisms can survive. Following this, sterilization of parts can be accomplished by immersion in either dilute bleach solutions, 70% ethanol, or commercial sanitizers [33].

### Long-term use considerations

The use of 3D printed hydroponics systems for food production raises important issues surrounding food safety, including microplastics leaching, degradation of parts due to UV exposure, heat, or humidity, and microbial growth within the system. Material selection is the most important factor when addressing these issues. Commercially, many hydroponics systems are made of food-grade polyvinyl chloride (PVC), which possesses adequate UV resistance, food safety, and mechanical strength characteristics for use in hydroponics. PETG is a common type of filament used in 3D printing, and is known for its improved UV resistance, temperature resistance, chemical resistance, food safety, and strength when compared to PLA, one of the most common types of 3D printing filaments [34–36]. PETG represents a viable alternative to PVC and PLA in hydroponics systems, and we recommend that users print this hydroponics system out of food-safe PETG filament for long-term use. Brass printing nozzles can also contain a small amount of lead and other heavy metals. Emerging evidence suggests that type and brand of 3D printing filament can influence the likelihood of microbial organisms [37]. We recommend using stainless steel printing nozzles for food safe applications and routinely sanitize 3D printed parts used in hydroponics systems. Additional treatment with a UV protective coating on exterior surfaces and sealing the system with food-grade epoxy may help minimize potential microplastic exposure, long-term degradation, and microbial ingress, but additional studies are needed to validate these approaches. However, UV radiation is not transmitted through greenhouse glass or most windows, so indoor use of these systems is unlikely to be affected by this type of light. We note that after two years of nearly constant use without any form of sealing, our components show minimal wear, signs of damage, or biofilm accumulation.

### System construction

Files for the single tower were downloaded from the NIH3D database (https://3d.nih.gov/entries/3DPX-021941), converted into gcode using PrusaSlicer, and printed on a Prusa MK3S+ 3D printer with PLA filament at 20% infill [25]. 3D printed objects were assembled into three individual single tower systems with three levels of 4-plant modules as described in our detailed protocol (dx.doi.org/10.17504/protocols.io.e6nvw4pw9lmk/v2; Supporting Information).

### Example use-case scenario: Plant material, salinity treatments, and harvest conditions

Spinach was selected to validate this system as it is generally considered to be a challenging crop to grow hydroponically, has similar growth characteristics to many other leafy greens and herbs, and has a relatively short life cycle. Four cultivars of spinach (Sunangel F1, Red Snapper F1, Auroch F1, and Flamingo Improved F1) were used in this study. Seeds were osmoprimed between two pieces of Whatman filter paper for three days in petri dishes containing 47.5:1 water: hydrogen peroxide solution. Petri dishes were placed in a Conviron PGW36 growth chamber (Conviron; Winnipeg, Canada) maintained at 20 °C and 55 ± 10% relative humidity, with 200 µmol/m^2^/s of photosynthetically active radiation for 12 hours over 10 days. After three days, seedlings were transferred onto felt wicks and installed into 2” neoprene collars and installed in plant cups. A detailed protocol for spinach germination can be found here: dx.doi.org/10.17504/protocols.io.5qpvodxebg4o/v3 [31]. Plants were randomly assigned into one of three blocks, which correspond with their height within the hydroponics towers they were installed into. Block 1 plants were on the lowest tower module, block 2 plants were on the middle tower module, and block 3 plants were on the top tower module. Plants were grown in quarter strength Hoagland’s No. 1 solution (0.6 dS/m) days 0–7 and switched to half-strength (1.2 dS/m) days 8–14 [29]. Full strength Hoagland’s No. 1 (2.4 dS/m) was provided days 14–21 until the start of treatments. For the remainder of the experiment (days 22–35), plants were provided either full strength Hoagland’s No. 1 nutrient solution (control), control + 20/10 mM NaCl/CaCl_2_ to an electrical conductivity (EC) of 4.6 dS/m (low salinity), or control + 20/10 mM NaCl/CaCl_2_ to an EC of 6.4 dS/m (mild salinity). The pH of all nutrient solutions was maintained between 5.5 and 6.0 using 0.2 M H_2_SO_4_ and 0.1 M KOH to lower or raise pH, respectively. After 35 days when plants had 6–8 mature leaves, tissue was harvested, weighed, and immediately frozen at −80 °C before processing. Dry weight was determined by lyophilizing pre-weighed frozen tissue over five days and subtracting the calculated moisture content from the original sample mass [38].

### Comparison to traditional deep water hydroponics

We grew the same varieties above (omitting Red Snapper F1) using a deep water culture hydroponics system. Red Snapper F1 was omitted due to the vastly different plant size and growth habit to avoid exaggerating the contribution of plant genetic background in this comparison. The deep water hydroponics system was comprised of a 20” x 15” x 7” bin with nine evenly spaced 2” holes on the lid. Two 12” long aquarium stones were added to the bin to ensure consistently high dissolved oxygen (>7.5 mg/L). Plants were cultured under the same environmental conditions as described above and harvested at 35 days when plants had 6–8 mature leaves.

### Statistical analysis and data visualization

Data were analyzed as a randomized complete block design with three treatments, four varieties, and three blocks. Analysis of variance was conducted in R statistical software followed by Tukey’s honestly significant difference test using the ‘car’ package [39,40]. Data were visualized using ggplot2 [41]. For the comparison between deep water culture hydroponics and our 3D printed hydroponics system, data were analyzed as a factorial design with two growing methods, three salinity treatments, and three varieties. Random effects modeling was conducted using the ‘lme4’ package [42].

## Expected results

### Fresh weight

**Fig 6:**
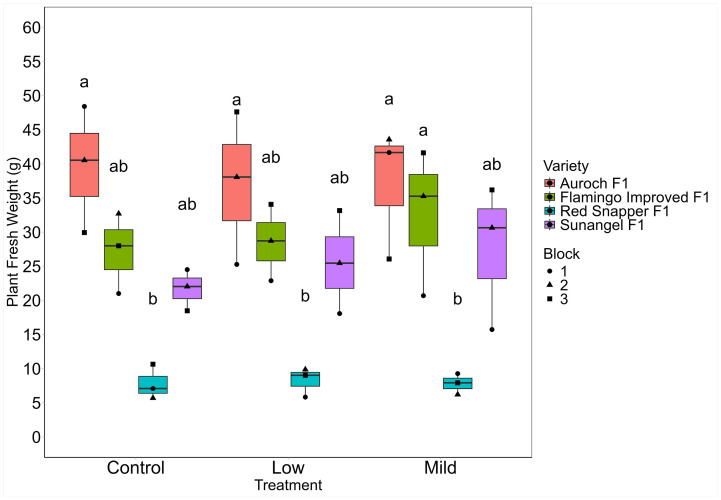
Fresh weight yield per plant by treatment and variety. Group letters from Tukey’s post-hoc test for significant differences (⍺ = 0.05) are displayed above each group.

There were no significant differences in mean fresh weight across any of the treatments when analyzing all varieties together (Fig 6; Table 8). Of the three treatments, mild salinity had the highest mean fresh weight at 26.23 grams, while the control treatment had the lowest at 24.09 grams. There was no significant treatment effect on fresh weight (p = 0.77), nor treatment-variety interaction (p = 0.95). However, the effect of variety on fresh weight was highly significant (p < 0.001), indicating that background genetics played a stronger role than salinity treatment on fresh weight. Auroch F1 had the highest mean fresh weight at 37.90 grams, while Red Snapper F1 had the lowest mean fresh weight at 7.95 grams. Flamingo F1 Improved and Sunangel F1 had mean fresh weight yields of 29.44 and 24.92 grams, respectively. Fresh weight yields are summarized in Table 9. Within each variety, there were no significant differences noted between treatment groups except for Flamingo Improved F1, which had a significant difference between the Low and Mild salinity conditions (Table 9; Fig 6). However, there was statistical significance for differences in fresh weight yield between varieties. Red Snapper F1 was significantly different from Auroch F1 in the control treatment, while in the Low salinity treatment it was significantly different from both Auroch F1 and Flamingo Improved F1. In the mild treatment, Sunangel F1 and Flamingo Improved F1 were significantly different.

**Table 9:** Mean fresh weight per plant (g) by treatment and variety.

	Treatment			
Variety	Average^a^	Control	Low	Mild
Auroch F1	37.91 ± 8.31a	39.63 ± 7.57a	36.99 ± 9.15a	37.10 ± 7.84a
Flamingo Improved F1	29.44 ± 6.73b	27.24 ± 4.81ab	28.56 ± 4.57ab	32.53 ± 8.76a
Sunangel F1	24.92 ± 6.74ab	21.68 ± 2.48ab	25.56 ± 6.16ab	27.52 ± 8.64ab
Red Snapper F1	7.95 ± 1.74c	7.81 ± 2.09b	8.25 ± 1.74b	7.80 ± 1.25b
Average	25.05 ± 12.64	24.08 ± 12.38a	24.83 ± 12.07a	26.23 ± 13.35a

All estimates reported means ± standard deviation as grams per plant. Similar letters indicate non-significance as determined by Tukey’s HSD (⍺ = 0.05).

^a^ Average values were run as a separate model examining either variety or treatment.

### Dry weight

**Fig 7:**
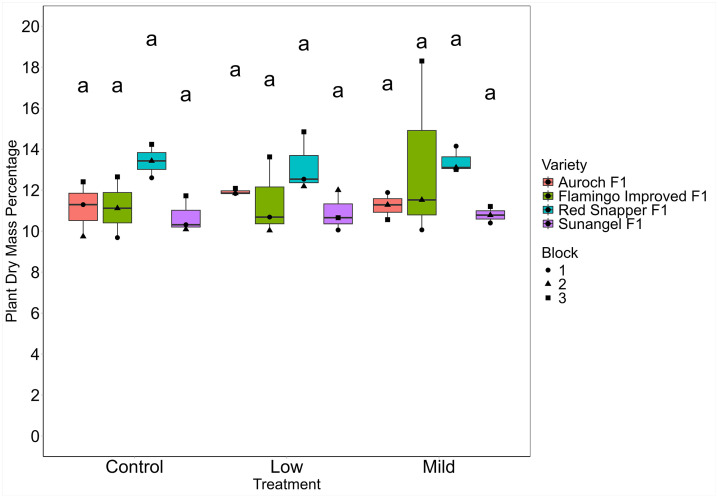
Dry mass percentage per plant by treatment and variety. Group letters from Tukey’s post-hoc test for significant differences (⍺ = 0.05) are displayed above each group.

There were no significant differences in dry weight percentage per plant between any of the varieties or treatments (Table 10; Fig 7). Dry weight percentages varied based on variety, with Red Snapper F1 having a slightly higher dry weight percentage per plant when compared to Auroch F1, Flamingo Improved F1, and Sunangel F1. Dry weight values for each variety and treatment are summarized in Table 10.

**Table 10:** Mean dry weight percentage per plant by treatment and variety.

Variety	Treatment			
	Average	Control	Low	Mild
Auroch F1	11.12 ± 0.79ab	11.15 ± 1.34a	11.92 ± 0.14a	11.24 ± 0.66a
Flamingo Improved F1	11.62 ± 2.55ab	11.15 ± 1.48a	11.45 ± 1.91a	13.29 ± 4.40a
Sunangel F1	10.80 ± 0.66b	10.71 ± 0.89a	10.90 ± 1.00a	10.79 ± 0.40a
Red Snapper F1	13.34 ± 0.84a	13.42 ± 0.82a	13.19 ± 1.44a	13.42 ± 0.63a
Average	11.72 ± 1.72	11.61 ± 1.43a	11.71 ± 1.36a	11.85 ± 2.19a

All estimates reported means ± standard deviation. Similar letters indicate non-significance as determined by Tukey’s HSD (⍺ = 0.05). Average values were run as a separate model examining either variety or treatment.

### General observations

Plants in the lower levels of the towers appeared to be smaller than their counterparts in the middle and upper levels of the towers. We hypothesized that this observation was due to shading effects from the plants above and distance from the light canopy, as the light source for this study was an overhead point source of lighting. We observed that the average photosynthetic photon flux density (PPFD) at the lowest, middle, and highest tower module was 355, 371, and 457 µmol/m^2^/s. Given a 12-hour photoperiod, this equates to a daily light integral (DLI) of 15.34, 16.04, and 19.74. Plants were assigned a block based on their position in each tower’s modules, with block 1 being the lowest level, block 2 being the middle level, and block 3 being the top level of each tower. The DLI of plants in block 3 was substantially higher due to being physically closer to the light fixture and not having shading from plants above. Although there were no significant differences in plant fresh weight by block (p = 0.11), block 3 plants had the highest fresh weights in 6 of the 12 unique combinations (Fig 6). Block did have a significant effect on dry weight percentage (p < 0.05). Block 3 plants were significantly different from block 1 plants (p < 0.05) and had the highest dry weights in 9 of the 12 unique salinity-variety combinations (Fig 7). This finding suggests that shading and distance from the light canopy could be a concern for researchers operating in growth chambers. However, this concern is not unique to our system as the light distribution of even the most sophisticated growth chambers is still controlled by the inverse square law. In more traditional, horizontal hydroponics systems, researchers often relocate their growing systems on a regular interval to evenly distribute environmental variation in light intensity. Still, shading can occur depending on the arrangement of plants in these systems. Taller plants such as rice or maize that are grown in growth chambers also experience major differences in light intensity depending on where light is being measured from within the canopy. To address this phenomenon, we recommend installing vertically mounted fixtures to more evenly provide light to growing plants. If possible, locating these systems in greenhouses or other locations where both natural and supplemental light can be more evenly distributed may help reduce inconsistencies.

Additionally, at the end of the experiment the nutrient solution flow rate was noted to be slightly lower for the control tower when compared to the low and mild salinity towers. During disassembly at harvest, root growth was observed in the filter and intakes of the submersible pumps in all bins. For longer duration experiments (e.g., > five weeks) we strongly recommend developing either a preventative or maintenance strategy to mitigate root ingress into nutrient solution pumps. The most effective mitigation strategy would be to utilize an externally mounted pump on top of the nutrient reservoir lid rather than using a submersible pump. Given the flexibility of our system, a spacer module could easily accommodate PVC fittings that could connect to internal tubing that draws nutrient solution from the reservoir into the pump and up the tower. Utilizing PVC or another opaque tubing is strongly recommended to minimize light interacting with the nutrient solution and promoting algal growth. A second reservoir with the pump inside that is remoted from the main system could also be considered. This modification would involve locating a sealed bin beneath the bench where the hydroponics tower is located and installing a standpipe inside connecting the bin to the reservoir. With sufficient diameter, the tubing connected to the submersible pump in the lower bin could pump nutrient solution up the standpipe and to the top of the tower. Nutrient solution would cascade down over the plant root systems, collected in the reservoir, and then return to the lower bin where the pump is located once the water level surpasses the height of the standpipe inside the reservoir. This solution would maintain a high level of dissolved oxygen and minimize the likelihood of roots getting near the intake of the pump. A final recommendation, albeit with less long-term utility, would be relocating the submersible pump into a corner of the reservoir to keep it further away from root intrusion.

### Comparison to traditional deep water culture hydroponics

We tested our 3D printed hydroponics system against a traditional, deep water hydroponics setup using the same experimental parameters except for Red Snapper F1 due to space limitations and its drastically smaller size/yield. Trends in fresh weight data for the remaining cultivars indicated that only variety was significantly different in this comparison (Table 11). We utilized random effects modeling to define how each model component (system type, salinity treatment, variety, and residual error) contributed to the total variance observed. System type explained only 0.02% of total variation while salinity treatment, variety, and residual error explained 2.47%, 67.42%, and 30.09% respectively. These data suggest that the growing system had virtually no impact on the conclusions drawn from the experiment. While additional replication may improve our power to detect differences between salinity treatments, our data suggest that in this context, responses are largely driven by plant genetic background. We recommend to researchers planning to use our 3D printed hydroponics suite for their experiments conduct pilot studies with their crop and treatments of interest to determine effect sizes, variance, and other critical experimental details prior to conducting large-scale studies.

**Table 11:** Analysis of variance outputs of the system comparison experiment comparing system type, treatment, and variety.

Model component	df^a^	Mean Square	F-value	p-value	Percent Variance Contributed
System Type	1	3.16	0.05	0.818	0.02%
Salinity Treatment	2	31.26	0.53	0.593	2.47%
Variety	2	1671.93	28.25	<0.001	67.42%
Residual	48	59.19			30.09%
Total	53				100%

^a^ df: degrees of freedom.

## Conclusions

Here, we describe and validate a publicly available 3D printed hydroponics suite for research and education. While hydroponic systems in general are well established, this work introduces a fully modular, additively manufactured design optimized for reproducibility, reconfiguration, and experimental use. Our systems are highly adaptable for a variety of applications and provide a cost-effective alternative to commercial hydroponics systems. Parts included in this suite can be utilized for any number of applications or modified by users to meet their own needs. In our experimental context, the 3D hydroponics system performed comparably to a traditional deep water culture setup. We encourage researchers to conduct pilot experiments to determine if any cultural modifications are needed prior to scaling-up to a full experiment. Future studies are needed to assess the long-term performance of these systems with a broader array of crops and to determine if food safety risks are any different compared to traditional hydroponics systems.

## Supporting information

S1 File A Protocols.IO document detailing the construction of the 3D printed hydroponics suite. (PDF)

S2 Table Fresh and dry weight percentage of plants harvested from the spinach salinity experiment. (CSV)

S3 Table Fresh weight of plants harvested for the system validation experiment. (CSV)

## References

[1] McNair MC, Cocioba SC, Pietrzyk P, Rife TW. Toward an open-source 3D-printable laboratory. Appl Plant Sci. 2024;12(1):e11562. doi: 10.1002/aps3.11562 38369980 PMC10873812

[2] Dzakovich M, Chlouber B. Multipurpose Universal Laboratory Tube Rack. NIH 3D; 2023. doi: 10.60705/3DPX/20367.1

[3] Dzakovich M, Chlouber B, Chandramouli S. Stackable 15 mL Tube Rack. NIH 3D; 2024. doi: 10.60705/3DPX/21167.2

[4] Dzakovich M, Chlouber B, Chandramouli S. Stackable 50 mL Tube Rack. NIH 3D; 2024. doi: 10.60705/3DPX/21168.2

[5] Lin K-Y, Hsiao H-S, Chang Y-S, Chien Y-H, Wu Y-T. The effectiveness of using 3D printing technology in STEM project-based learning activities. Eurasia J Math Sci Tech. 2018;14(12). doi: 10.29333/ejmste/97189

[6] Cheng L, Antonenko P “Pasha,” Ritzhaupt AD, MacFadden B. Exploring the role of 3D printing and STEM integration levels in students’ STEM career interest. Brit J Educational Tech. 2021;52(3):1262–78. doi: 10.1111/bjet.13077

[7] Chien YH, Chu PY. The different learning outcomes of high school and college students on a 3D-printing STEAM engineering design curriculum. Int J Sci Math Educ. 2018;16:1047–64. doi: 10.1007/s10763-017-9832-4

[8] Abu Khurma O, Ali N, Swe Khine M. Exploring the impact of 3D printing integration on STEM attitudes in elementary schools. Cont Ed Technol. 2023;15(4):ep458. doi: 10.30935/cedtech/13568

[9] Kaiser E, Kusuma P, Vialet-Chabrand S, Folta K, Liu Y, Poorter H. Vertical farming goes dynamic: optimizing resource use efficiency, product quality, and energy costs. Front Sci. 2024;2. doi: 10.3389/fsci.2024.1411259

[10] Mitchell CA. History of controlled environment horticulture: Indoor farming and its key technologies. 2022. doi: 10.21273/HORTSCI16159-21

[11] Grusak MA. Intrinsic stable isotope labeling of plants for nutritional investigations in humans. J Nutrition Biochem. 1997;8(4):164–71. doi: 10.1016/s0955-2863(97)00017-x

[12] Poulet L, Massa GD, Morrow RC, Bourget CM, Wheeler RM, Mitchell CA. Significant reduction in energy for plant-growth lighting in space using targeted LED lighting and spectral manipulation. Life Sci Space Res. 2014;2:43–53. doi: 10.1016/j.lssr.2014.06.002

[13] Sheibani F, Bourget M, Morrow RC, Mitchell CA. Close-canopy lighting, an effective energy-saving strategy for overhead sole-source LED lighting in indoor farming. Front Plant Sci. 2023;14:1215919. doi: 10.3389/fpls.2023.1215919 37575942 PMC10413563

[14] Sheibani F, Runkle E, Mitchell CA. Interaction of the far-red radiation intensity and CO2 concentration during early indoor production stages of red leaf lettuce. 2024. doi: 10.21273/JASHS05434-24

[15] Almerino J, Peria JN. Enhancing biology education through hydroponics: a practical approach in high school classes. TheQUEST: J Multidiscip ResDev. 2024;3(1). doi: 10.60008/thequest.v3i1.190

[16] Patchen A, Aeschlimann A, Vera-Cruz A, Kamath A, Jose D, DeLisi J, et al. Seeding the future: blending urban gardening with community outreach and STEM learning. Conn Sci Learn. 2017;1:12420467. doi: 10.1080/24758779.2017.12420467

[17] Reed H, Stanton A, Sood A, Knowles M. Hydroponics Playground Garden, “Playponics”; designing integrated sustainability and STEM education through play. Mumbai, India: IDC School of Design, IIT Bombay; 2021.

[18] Guasti L, Niewint-Gori J. Looking for New Ways to Grow: A Hydroponic Indoor Garden at School to Improve STEM Education and 21st Century Skills. In: ICERI Proceedings. 2018. pp. 2631–40. doi: 10.21125/iceri.2018.1583

[19] Morris J, Neustadter A, Zidenberg-Cherr S. First-grade gardeners more likely to taste vegetables. CalAg. 2001;55:43–6. doi: 10.3733/ca.v055n01p43

[20] Heim S, Stang J, Ireland M. A garden pilot project enhances fruit and vegetable consumption among children. J Am Diet Assoc. 2009;109(7):1220–6. doi: 10.1016/j.jada.2009.04.009 19559139

[21] Kwok SWH, Wu CST, Tong HT, Ho CN, Leung KL, Leung YCP, et al. Effects of the school-based integrated health promotion program with hydroponic planting on green space use and satisfaction, dietary habits, and mental health in early adolescent students: a feasibility quasi-experiment. Front Public Health. 2021;9:740102. doi: 10.3389/fpubh.2021.740102 34631651 PMC8498580

[22] Kim S-O, Park S-A. Garden-based integrated intervention for improving children’s eating behavior for vegetables. Int J Environ Res Public Health. 2020;17(4):1257. doi: 10.3390/ijerph17041257 32075303 PMC7068610

[23] Parra X, Jiménez N, Simarro C, Pérez E, Monteagudo V, Grané J, et al. Enhancing Student Learning Through Aeroponics and PBL: A Steam Collaborative Project. In: EDULEARN Proceedings. 2024. pp. 4674–82. doi: 10.21125/edulearn.2024.1153

[24] Thompson KR, Webster CD, Pomper KW, Krall RM. Use of aquaponics project-based environments to improve students’ perception of science, technology, engineering, and mathematics (STEM) disciplines and career pathways. Interdiscip J Environ Sci Educ. 2023;19:e2309. doi: 10.29333/ijese/13102

[25] Chandramouli S, Shaw E, Dzakovich M. Single tower modular 3D printed hydroponics system. NIH 3D; 2025. doi: 10.60705/3DPX/21941.3 PMC13127910 42054324

[26] Chandramouli S, Shaw E, Dzakovich M. Double tower modular 3D printed hydroponics system. NIH 3D; 2025. doi: 10.60705/3DPX/21942.2 PMC13127910 42054324

[27] Lovett B. Modular Hydroponic Tower Garden by boundarycondition - Thingiverse. 2020 [cited 25 Jun 2025]. Available from: https://www.thingiverse.com/thing:3405964

[28] Hoagland DR, Arnon DI. The water-culture method for growing plants without soil. Calif Agr Expt Sta Cir. 1950.

[29] Hernandez A, Dzakovich M. Making Hoagland’s Complete Nutrient Solution for Hydroponics V.2. 2026 [cited 13 Mar 2026]. Available from: https://www.protocols.io/view/making-hoagland-39-s-complete-nutrient-solution-fo-36wgqpxp5vk5/v2

[30] Langenfeld NJ, Bugbee B. Germination and seedling establishment for hydroponics: The benefit of slant boards. PLoS One. 2022;17(10):e0275710. doi: 10.1371/journal.pone.0275710 36197903 PMC9534409

[31] Hernandez A, Chandramouli S, Dzakovich M. Spinach Germination Protocol V.3. 2026 [cited 13 Mar 2026]. Available from: https://www.protocols.io/view/spinach-germination-protocol-5qpvodxebg4o/v3

[32] Chandramouli S, Dzakovich M. Slant Board Mounting Fixture for Seed Germination. NIH 3D; 2024. doi: 10.60705/3DPX/21396.1

[33] Thomas M, Amin A, Seibi A, Jafar I. Innovations in sanitization for 3D-printed parts in medical and critical applications. 2025. doi: 10.13140/RG.2.2.25371.99361

[34] Durga Rajesh KV, Ganesh N, Yaswanth Kalyan Reddy R, Mishra H, Teja Naidu TMVPS. Experimental research on the mechanical characteristics of fused deposition modelled ABS, PLA and PETG specimens printed in 3D. Mater Today: Proceedings. 2023. doi: 10.1016/j.matpr.2023.06.343

[35] Suh D, Hockett Sherlock S, Dukes KC, Perencevich EN, Marra AR. Impact of UV-C on material degradation: a scoping literature review. Antimicrob Steward Healthc Epidemiol. 2025;5(1):e199. doi: 10.1017/ash.2025.10114 40927244 PMC12415791

[36] Amza CG, Zapciu A, Baciu F, Vasile MI, Nicoara AI. Accelerated aging effect on mechanical properties of common 3D-printing polymers. Polymers (Basel). 2021;13(23):4132. doi: 10.3390/polym13234132 34883635 PMC8659210

[37] Massa G, Torres J, Hummerick M, Montgomery E, Mott J, Dreschel T, et al. 3D Printed Materials Characterization for Rapid Prototyping and Plant Growth. 2024. Available from: https://ntrs.nasa.gov/citations/20240001601

[38] Hernandez A, Dzakovich M. Freeze Drying Plant and Food Samples. 2025 [cited 13 Mar 2026]. Available from: https://www.protocols.io/view/freeze-drying-plant-and-food-samples-261gek12jg47/v1

[39] R Development Core Team. R: A language and environment for statistical computing. Vienna, Austria; 2018.

[40] Fox J, Weisberg S. An {R} Companion to Applied Regression. 3rd ed. Thousand Oaks, CA: Sage; 2019.

[41] Wickham H. ggplot2 Elegant Graphics for Data Analysis. New York, NY: Springer; 2016. doi: 10.1007/978-0-387-98141-3

[42] Bates D, Mächler M, Bolker B, Walker S. Fitting linear mixed-effects models using lme4. J Stat Softw. 2015;67:1–48. doi: 10.18637/jss.v067.i01

